# Comparison of renal remission and relapse‐free rate in initial‐ and delayed‐onset lupus nephritis

**DOI:** 10.1111/1756-185X.14228

**Published:** 2021-10-11

**Authors:** Eiji Suzuki, Makiko Yashiro‐Furuya, Jumpei Temmoku, Yuya Fujita, Naoki Matsuoka, Momoko Hazama, Tomoyuki Asano, Shuzo Sato, Hiroko Kobayashi, Hiroshi Watanabe, Takashi Kanno, Kiyoshi Migita

**Affiliations:** ^1^ Department of Rheumatology Ohta‐Nishinouchi Hospital Fukushima Japan; ^2^ Department of Rheumatology Fukushima Medical University School of Medicine Fukushima Japan; ^3^ Ohga Clinic Fukushima Japan

**Keywords:** immunosuppressive agents, lupus nephritis, prednisolone, recurrence, systemic lupus erythematosus

## Abstract

**Introduction:**

Lupus nephritis (LN) is a major manifestation of systemic lupus erythematosus (SLE) which contributes to significant morbidity and mortality. It is unclear whether the timing of LN onset influences renal outcome. This study aimed to investigate differences in clinical features—particularly the relapse‐free rate—in remission duration from induction therapies for LN and the onset timing of LN after the development of SLE.

**Methods:**

We enrolled 66 LN patients from January 2004 to March 2020. We collected the following: demographic data, laboratory data, renal histology data, and LN induction therapy data. Renal remission and relapse‐free rates were calculated for each group.

**Results:**

Patients were first divided into early remission group (achieved renal remission in <12 months [n = 44]) and others (n = 22). There were no significant differences in clinical data, treatments, and relapse‐free rate of LN. Patients were then divided into initial‐onset LN (<12 months after development of SLE [n = 49]) and delayed‐onset LN (≥12 months after development of SLE [n = 17]). Kaplan–Meier analysis showed that the relapse‐free rate was significantly higher in all patients with initial‐onset LN than those with delayed‐onset LN (*P* = .0094).

**Conclusion:**

The relapse‐free rate was significantly higher in the initial‐onset LN group than the delayed‐onset LN group of patients with LN of various histopathological backgrounds. These data suggest that delayed‐onset LN is a risk factor for the relapse of LN.

## 1 INTRODUCTION

Systemic lupus erythematosus (SLE) is an autoimmune disease characterized by a loss of self‐tolerance and formation of nuclear autoantigens and immune complexes, resulting in inflammation of multiple organs.^1^ Lupus nephritis (LN) is a major manifestation of SLE that contributes to significant morbidity and mortality.^2^ Patients with LN have higher mortality rates than SLE patients without LN.^3^ From 2012 to 2013, clinical guidelines for LN were reported from the American College of Rheumatology (ACR),^4^ Kidney Disease Improving Global Outcome,^5^ joint European League Against Rheumatism (EULAR), European Renal Association (ERA), European Dialysis and Transplant Association (EDTA),^6^ and Asian Lupus Nephritis Network.^7^ EULAR updated the management recommendation for SLE in 2019.^8^ Those clinical guidelines recommend performing a renal biopsy in order to obtain renal histology unless strongly contraindicated. Also, treatment should be based on the type of LN, as classified by the International Society of Nephrology/Renal Pathology Society (ISN/RPS) criteria.^9^, ^10^ Additionally, in induction therapies for classes Ⅲ or Ⅳ LN, the use of mycophenolate mofetil (MMF) or intravenous cyclophosphamide (IVCY), along with glucocorticoids, was recommended based on various clinical trials including the Aspreva Lupus Management Study.^11^ Further, it has been reported that renal response after 6 months of treatment with IVCY can predict long‐term renal outcomes based on data obtained in the Euro‐Lupus Nephritis Trial.^12^ Reports also suggest that, in Japan, achieving early renal remission of classes Ⅲ or Ⅳ LN using glucocorticoids with immunosuppressants might predict good outcomes, such as reduced organ damage and a low incidence of disease flare.^13^, ^14^

On the other hand, some patients develop SLE and LN simultaneously, while others develop LN after an SLE diagnosis without renal involvement. According to several reports, the number of SLE patients who developed LN later was fewer compared to those who were diagnosed with LN simultaneously or within a few years.^15^, ^16^, ^17^, ^18^ It is unclear whether the timing of the onset of LN influences renal outcome. Reports from Ugolini‐Lopes et al^19^ and Dlifino et al^20^ showed that no major differences were noted when disease profile or treatment outcome of early‐ and late‐onset LN were compared. On the other hand, several reports from Japan showed that SLE patients with early‐onset LN had better renal outcomes when compared to those with late delayed‐onset LN.^18^, ^21^, ^22^ Because the results were different, it is unclear how the timing of LN onset affects its pathophysiology. Although a renal biopsy is recommended for diagnosing and treating LN, it is difficult to achieve in patients at risk of bleeding, in poor general condition, or who refuse it. In clinical settings, it is often difficult to treat and predict the prognosis of patients with LN who have not been histologically diagnosed.

This study investigated SLE patients with LN at Ohta‐Nishinouchi Hospital and Fukushima Medical University Hospital to assess if there were differences in clinical, serologic profile, treatments, and relapse‐free rate of 2 groups of patients. One group involved duration of remission of LN from induction therapies (<6 months and ≥6 months), and the other involved onset timing of LN after SLE development (<12 months after SLE development or ≥12 months).

## 2 METHODS

### 2.1 Patients

Medical records of LN patients at Ohta‐Nishinouchi Hospital and Fukushima Medical University Hospital who suffered from the disease for 1 or more years—from January 2004 to March 2020—were reviewed. SLE was diagnosed according to the ACR classification criteria of SLE,^23^ while LN was diagnosed according to pathological findings obtained from renal biopsy. In lieu of renal biopsy, diagnosis of LN was made using the criteria of renal disorder aspect of the ACR classification criteria of SLE.^23^ The study population consisted of 66 patients (55 female and 11 male). Complete renal response (CR) and partial renal response (PR) were defined on the basis of EULAR/ERA‐EDTA recommendations for LN,^6^ with CR defined as a urine protein/creatinine ratio (UPCR) <50 mg/mmol and normal or near‐normal (within 10% of normal glomerular filtration rate [GFR] if previously abnormal) renal function, and PR defined as a ≥ 50% reduction in proteinuria and normal or near‐normal GFR. As previously described by Hanaoka et al, 0.5 g/g creatinine was considered equivalent to UPCR 50 mg/mmol.^14^ This study defined renal remission as PR including CR if achieved. Renal relapse was defined as loss of CR or PR status after achieving CR or PR. We divided patients into 2 groups based on time of renal remission and time of LN development from SLE onset.

### 2.2 Data collection

Patients' baseline characteristics were collected at the time of diagnosis with SLE and LN. Demographic data included age at onset of SLE and LN, gender, disease duration of SLE and LN, time lag between onset of SLE and LN, observation period of the patients, patient scores on the SLE disease activity index 2000 (SLEDAI‐2K),^24^ one of the disease activity scoring systems for SLE, at the time of diagnosis with LN, and comorbidities of anti‐phospholipid syndrome (APS). Laboratory data collected at the onset of LN included total protein, albumin, serum creatinine, estimated GFR (eGFR), complement 3 (C3), complement 4 (C4), UPCR, and positivity of anti‐double‐strand‐DNA (anti‐ds‐DNA) antibodies. Anti‐ds‐DNA antibodies were measured by radioimmunoassay, enzyme‐linked immunosorbent assay, or fluorescent enzyme immunoassay methods. Obtained renal tissues were diagnosed with LN according to the World Health Organization criteria or ISN/RPS classification of LN if renal biopsy was performed. Data on induction therapy of LN were also collected from medical records.

### 2.3 Statistical analysis

Continuous values are shown as median and interquartile range (IQR). A nonparametric Mann–Whitney *U* test was used for inter‐group comparisons of multiple variables. Fisher's exact test was used to investigate a possible association between each variable. Kaplan–Meier method was used to calculate the rate of remission and relapse‐free rate, while a log‐rank test was used to assess differences between the 2 groups. GraphPad Prism 5 software (GraphPad Software, San Diego, CA) was used to perform all of the statistical analyses. The significance level was set at *P* <.05.

### 2.4 Ethics approval and consent to participate

The study was approved by the Ethics Committee of Fukushima Medical University (No. 30155).

## 3 RESULTS

### 3.1 Patient characteristics

The demographic and disease‐related features of the enrolled 66 patients are as follows (Table 1). The majority of the patients were female (83.3%). The median age at onset of LN was 31.0 years (IQR 25.0‐44.0 years), the disease duration of SLE was 81.5 months (IQR 37.3‐143.0 months), observation period of the patients was 56.0 months (IQR 30.0‐122.3), and SLEDAI‐2K score at the time of onset of LN was 10.0 (IQR 10.0‐18.0). Renal biopsy was performed on 45 (68.2%) patients. Thirty‐eight (57.6%) patients were treated with intravenous methyl‐prednisolone (mPSL) pulse therapy, 17 (25.6%) with IVCY, 8 (12.1%) with tacrolimus (TAC), 9 (13.6%) with MMF, and 3 (4.5%) with TAC plus MMF for induction therapy. Sixty‐five patients (98.5%) achieved renal remission during the study period.

**TABLE 1:** Baseline characteristics of patients

Characteristics		Overall (n = 66)
Female, n (%)		55 (83.3)
Age at onset of LN, y, median [IQR]		31.0 [25.0‐44.0]
Disease duration of LN, mo, median [IQR]		81.5 [37.3‐143.0]
Observation period, mo, median [IQR]		56.0 [30.0‐122.3]
SLEDAI‐2K score at the onset of LN, median [IQR]		15.0 [10.0‐18.0]
Total protein, mg/dL, median [IQR]		6.7 [5.9‐7.6]
Albumin, mg/dL, median [IQR]		3.2 [2.5‐3.7]
Creatinine, mg/dL, median [IQR]		0.70 [0.59‐0.93]
eGFR, mL/min, median [IQR]		79.3 [59.5‐93.9] (n = 61)
C3, mg/dL, median [IQR]		42.0 [29.1‐70.5] (n = 65)
C4, mg/dL, median [IQR]		5.0 [3.2‐11.0] (n = 65)
Urine protein, g/g creatinine, median [IQR]		1.34 [0.61‐3.12] (n = 56)
Anti‐dsDNA antibody, n (%)		52 (78.8)
Antiphospholipid antibody syndrome, n (%)		5 (7.6)
Renal histopathology of lupus nephritis	II	4
	III	8
	IV	11
	V	10
	II +V	1
	III +V	6
	IV +V	5
	N/A	21
Induction therapy	Intravenous mPSL pulse (%)	38 (57.6)
	CYC (%)	17 (25.8)
	TAC (%)	8 (12.1)
	MMF (%)	9 (13.6)
	MMF +TAC (%)	3 (4.5)

Abbreviations: C3, complement 3; C4, complement 4; CYC, cyclophosphamide; eGFR, estimated glomerular filtration rate; IQR, interquartile range; LN, lupus nephritis; MMF, mycophenolate mofetil; mPSL, methylprednisolone; N/A, not available; SLEDAI‐2K, systemic lupus erythematosus disease activity index 2000; TAC, tacrolimus.

### 3.2 Comparison of LN patients according to time of renal remission

**FIGURE 1:**
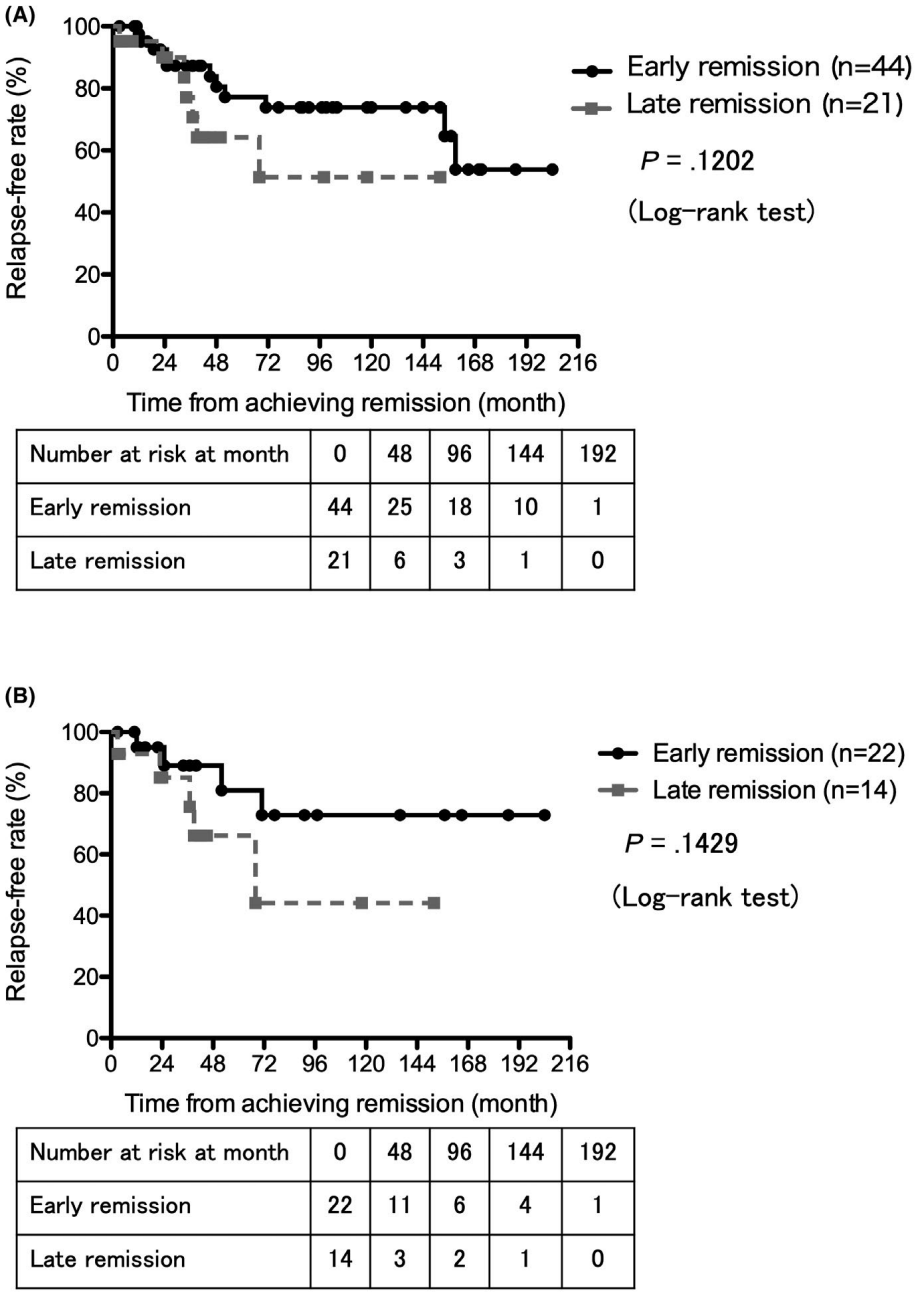
The relapse‐free rate from achieving renal remission between early and late remission patients of lupus nephritis. A: There is no significant difference in the relapse‐free rate between early (n = 44) and late (n = 21) remission patients of lupus nephritis (log‐rank test, *P* =.1202). B: There is no significant difference in the relapse‐free rate between early (n = 22) and late (n = 14) remission patients of lupus nephritis treated with prednisolone plus immunosuppressants (log‐rank test, *P* =.1429)

Enrolled SLE patients were initially divided into 2 groups based on the time of renal remission. Since the Euro‐Lupus Nephritis Trial results showed that early response to therapy at 6 months was the best predictor of good long‐term renal outcomes,^12^ we categorized the patients as follows. Patients who achieved renal remission in <6 months were defined as early remission, and others (ie, patients who achieved renal remission at ≥6 months and those who did not achieve remission) were defined as late remission. The demographic, disease‐related features, renal histopathology, and induction therapies were summarized in each group and compared (Table 2): 44 patients (66.7%) achieved early remission, while 22 (33.3%) achieved late remission. There were no significant differences between the 2 groups regarding disease‐related features at baseline. No significant difference was observed in terms of the observation period between the 2 groups, and 1 patient in the late remission group did not achieve partial remission during the observation period. There were no significant differences regarding kidney histopathology of LN, although 14 patients of early remission and 7 patients of late remission did not have renal biopsies conducted for various reasons. In induction therapies for LN, more patients were treated with mPSL pulse therapy in the early remission group, but there was no significant difference. A Kaplan–Meier analysis showed that the relapse‐free rate after induction therapies was not significantly different between the early and late remission groups (*P* = .1202, log‐rank test) (Figure 1A). Regarding medication of induction therapies, there were no significant differences in the relapse‐free rate in the 2 groups treated with only PSL including mPSL pulse therapy (early remission 13/19 [68.4%] vs late remission 1/1 [100%]), and treated with PSL (including mPSL pulse therapy) plus immunosuppressants including IVCY, TAC, MMF, and TAC plus MMF (early remission: n = 22, late remission: n = 14, *P* =.1429, log‐rank test) (Figure 1B).

**TABLE 2:** Comparison of the characteristics of LN patients with early remission and late remission (including non‐remission)

Characteristics		Early remission (n = 44)	Late remission (n = 22)	*P* value*
Female, n (%)		38 (86.3)	17 (77.3)	.4849
Age at onset of LN, y, median [IQR]		30.0 [24.3‐43.8]	28.5 [23.0‐44.3]	.8917
Disease duration of LN, mo, median [IQR]		91.0 [35.8‐152.8]	66.5 [37.3‐133.5]	.6732
Observation period, mo, median [IQR]		75.0 [27.8‐136.5]	52.5 [31.5‐100.5]	.5050
SLEDAI‐2K score at the onset of LN, median [IQR]		15.0 [10.3‐18.0]	15.5 [9.8‐18.3]	.9077
Total protein, mg/dL, median [IQR]		6.7 [5.9‐7.8]	6.8 [6.0‐7.1]	.6732
Albumin, mg/dL, median [IQR]		3.0 [2.5‐3.6]	3.2 [2.6‐3.7]	.4454
Creatinine, mg/dL, median [IQR]		0.70 [0.59‐0.88]	0.77 [0.62‐1.02]	.1257
eGFR, mL/min, median [IQR]		83.5 [64.7‐99.9] (n = 40)	70.8 [51.5‐91.8] (n = 21)	.0835
C3, mg/dL, median [IQR]		42.6 [28.0‐63.0] (n = 43)	38.7 [30.9‐73.3]	.8842
C4, mg/dL, median [IQR]		5.0 [3.0‐13.0] (n = 43)	5.0 [3.6‐8.6]	.8241
Urine protein, g/g creatinine, median [IQR]		1.28 [0.57‐2.82] (n = 37)	1.40 [0.90‐3.68] (n = 19)	.5738
Anti‐dsDNA antibody, n (%)		35 (79.5)	17 (77.3)	1.0000
Antiphospholipid antibody syndrome, n (%)		3 (6.8)	2 (9.1)	1.0000
Renal histopathology of lupus nephritis	II	3	1	1.0000
	III	6	2	1.0000
	IV	5	6	.1594
	V	8	2	.4755
	II +V	0	1	.3333
	III +V	5	1	.6549
	IV +V	3	2	1.0000
	N/A	14	7	1.0000
Induction therapy	Intravenous mPSL pulse (%)	29 (65.9)	9 (40.9)	.0674
	CYC (%)	11 (25.0)	6 (27.3)	1.0000
	TAC (%)	5 (11.4)	3 (13.6)	1.0000
	MMF (%)	6 (13.6)	3 (13.6)	1.0000
	MMF +TAC (%)	0 (0)	3 (13.6)	N/A

Abbreviations: C3, complement 3; C4, complement 4; CYC, cyclophosphamide; eGFR, estimated glomerular filtration rate; IQR, interquartile range; LN, lupus nephritis; MMF, mycophenolate mofetil; mPSL, methylprednisolone; N/A, not available; SLEDAI‐2K, systemic lupus erythematosus disease activity index 2000; TAC, tacrolimus.

*P values were determined using nonparametric Mann–Whitney *U* test or Fisher's exact test.

### 3.3 Comparison of LN patients according to timing of LN development from SLE onset

**FIGURE 2:**
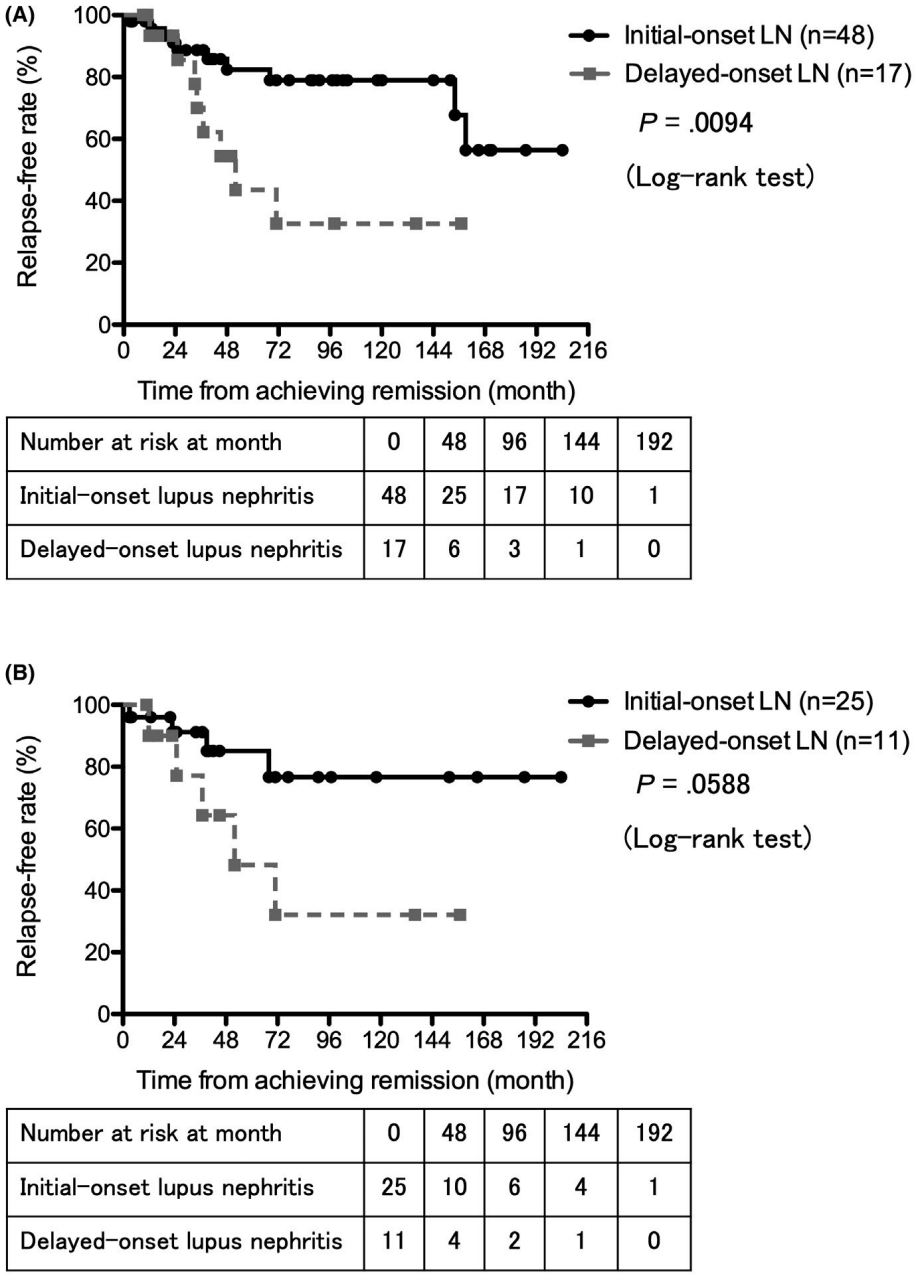
The relapse‐free rate from achieving renal remission between patients of initial‐ and delayed‐onset lupus nephritis (LN). A: There is a significant difference in the relapse‐free rate between patients of initial‐ (n = 48) and delayed‐onset LN (n = 17) (log‐rank test, *P* =.0094). B: The relapse‐free rate of patients of initial‐onset LN (n = 25) treated with prednisolone plus immunosuppressants was higher when compared to patients of delayed‐onset LN (n = 11) (*P* = .0588, log‐rank test)

Enrolled SLE patients were then divided into another 2 groups based on timing of LN development from SLE onset. Jacobsen et al^16^ and Seligman et al^17^ demonstrated that renal manifestation in most SLE patients was observed within a year, and therefore, we divided the patients as follows. Patients who were diagnosed with SLE and LN simultaneously, or LN within <12 months of diagnosis with SLE, were defined as initial‐onset LN, and others (ie, patients who were diagnosed with LN after ≥12 months of diagnosis with SLE, and who had already achieved remission of SLE before being diagnosed with LN) were defined as delayed‐onset LN (Table 3). There were 49 patients (74.2%) with initial‐onset LN, and 17 (25.8%) with delayed‐onset LN. Median duration from onset of SLE to onset of LN in delayed‐onset LN patients was 41.0 months. No significant difference in observation period was observed between the 2 groups, and 1 patient in the initial‐onset LN group did not achieve partial remission during the observation period. Of the disease‐related features at baseline, higher serum albumin levels (*P* = .0086) and higher serum C3 levels (*P* = .0477) were significantly related to delayed‐onset LN, and significantly higher SLEDAI‐2K scores at the time of onset of LN (*P* =.0069) were also observed in the delayed‐onset LN group. There were no significant differences regarding histopathology of the LN kidney, although 17 patients of initial‐onset LN and 4 patients of delayed‐onset LN did not have renal biopsies for various reasons. With respect to induction therapies for LN, more patients were treated with mPSL pulse therapy in initial‐onset LN compared with delayed‐onset LN (*P* =.0461). Among 49 patients, 48 (98.0%) in initial‐onset LN, and 17 of 17 (100%) patients in delayed‐onset LN groups achieved renal remission during the observation period. Kaplan–Meier analysis showed that the renal remission rate was not significantly different between initial‐onset LN and delayed‐onset LN (data not shown). We then analyzed the relapse‐free rate of initial‐onset LN and delayed‐onset LN after induction of LN. Thirty‐eight of the 48 patients (79.2%) in initial‐onset LN, and 9 of 17 patients (52.9%) in delayed‐onset LN did not experience renal relapse during the observation period. Kaplan–Meier analysis showed that the relapse‐free rate was significantly higher in patients of initial‐onset LN when compared to those of delayed‐onset LN (*P* = .0094, log‐rank test) (Figure 2A). Regarding medication for induction therapies, there was no significant difference in the relapse‐free rate in the 2 groups treated with only PSL including mPSL pulse therapy (initial‐onset LN 13/18 [72.2%] vs delayed‐onset LN 1/2 [50%]). Also, the relapse‐free rates of initial‐onset LN patients treated with PSL (including mPSL pulse therapy) plus immunosuppressants—including IVCY, TAC, MMF, and TAC plus MMF—tended to be higher when compared to those of delayed‐onset LN patients (initial‐onset LN: n = 25, delayed‐onset LN: n = 11, *P* = .0588, log‐rank test) (Figure 2B).

**TABLE 3:** Comparison of the characteristics of patients with initial‐onset LN and delayed‐onset LN

Characteristics		Initial‐onset LN (n = 49)	Delayed‐onset LN (n = 17)	*P* value^†^
Female, n (%)		41 (83.7)	14 (82.4)	1.0000
Age at onset of LN, y, median [IQR]		30.0 [24.5‐44.5]	33.0 [27.5‐40.5]	.3826
Disease duration of LN, mo, median [IQR]		90.0 [38.0‐151.5]	67.0 [32.0‐138.0]	.8834
Duration between onset of SLE and onset of LN, mo, median [IQR]			41.0 [26.0‐106.5]	
Observation period, mo, median [IQR]		74.0 [33.0‐126.5]	46.0 [27.5‐80.5]	.2588
SLEDAI‐2K score at onset of LN, median [IQR]		16.0 [13.0‐19.0]	10.0 [8/0‐16.5]	.00069*
Total protein, mg/dL, median [IQR]		6.7 [5.9‐7.8]	6.7 [6.0‐7.0]	.8487
Albumin, mg/dL, median [IQR]		3.0 [2.4‐3.5]	3.7 [3.0‐3.9]	.0086*
Creatinine, mg/dL, median [IQR]		0.71 [0.59‐0.97]	0.67 [0.63‐0.74]	.5377
eGFR, mL/min, median [IQR]		78.6 [55.2‐93.1] (n = 44)	83.0 [66.8‐98.7]	.4545
C3, mg/dL, median [IQR]		38.0 [27.5‐63.0]	54.6 [37.5‐76.0] (n = 16)	.0477*
C4, mg/dL, median [IQR]		5.0 [3.0‐12.3]	6.6 [3.7‐9.9] (n = 16)	.6417
Urine protein, g/g creatinine, median [IQR]		1.28 [0.57‐2.97] (n = 41)	1.78 [0.7.0‐3.64] (n = 15)	.5476
Anti‐dsDNA antibody, n (%)		37 (75.5)	15 (88.2)	.3272
Antiphospholipid antibody syndrome, n (%)		4 (8.2)	1 (5.9)	1.0000
Renal histopathology of lupus nephritis	II	3	1	1.0000
	III	7	1	.6689
	IV	8	3	1.0000
	V	7	3	.7093
	II +V	1	0	1.0000
	III +V	3	3	.1722
	IV +V	3	2	.5970
	N/A	17	4	.5485
Induction therapy	Intravenous mPSL pulse (%)	32 (65.3)	6 (35.3)	.0461*
	CYC (%)	14 (28.6)	3 (17.6)	.5247
	TAC (%)	4 (8.2)	4 (23.5)	.1889
	MMF (%)	6 (12.2)	3 (17.6)	.6844
	MMF +TAC (%)	2 (4.1)	1 (5.9)	1.0000

Abbreviations: C3, complement 3; C4, complement 4; CYC, cyclophosphamide; eGFR, estimated glomerular filtration rate; IQR, interquartile range; LN, lupus nephritis; MMF, mycophenolate mofetil; mPSL, methylprednisolone; N/A, not available; SLEDAI‐2K, systemic lupus erythematosus disease activity index 2000; TAC: tacrolimus.

^†^ *P* values were determined using nonparametric Mann–Whitney *U* test or Fisher's exact test.

*indicates *P* <.05.

## 4 DISCUSSION

This study demonstrated that the relapse‐free rate of LN in delayed‐onset LN patients was significantly low when compared to that of initial‐onset LN patients. We also showed that delayed‐onset LN patients who were administered immunosuppressants experienced more relapses of LN when compared to initial‐onset LN patients who were administered immunosuppressants. We could not show those differences in the study of time of remission (early remission and late remission) in our patients.

In delayed‐onset LN patients, serum albumin and C3 were significantly high when compared to initial‐onset LN patients. However, there was no difference regarding the distribution of renal histopathology in patients who underwent renal biopsy. In addition, there was no difference in induction therapies between initial‐onset LN and delayed‐onset LN, except that patients who were prescribed steroid pulse therapy were significantly higher in the initial‐onset LN group. Delayed‐onset LN patients had immunosuppressive treatments for SLE before LN development. In our LN patients, there were no delayed‐onset LN patients who developed neuropsychiatric SLE before development of LN. With the exception of only a few delayed‐onset LN patients who received immunosuppressants, most delayed‐onset LN patients were administered a low to moderate amount of glucocorticoid for mild to moderate symptoms—such as arthritis and rash—before development of LN. Therefore, although the nature of nephritis is possibly associated with poor immune complex activity in delayed‐onset LN, it was considered that the reason serum albumin and C3 levels were high in delayed‐onset LN patients was that the immune complex activity of SLE improved following prior treatments, as suggested by the lower SLEDAI‐2K score in the delayed‐onset LN patients. It was also considered that delayed‐onset LN patients were being watched closer, therefore, their proteinuria was caught earlier than the initial‐onset LN patients. Therefore, urinary loss of protein and increased complement consumption might have been going on for weeks or months before initial‐onset LN patients present for clinical care and are diagnosed with LN which would result in the reduction of serum albumin and C3. However, although laboratory data of delayed‐onset LN patients tended to be better than those of initial‐onset LN patients because of preceding treatments, the relapse‐free rate was lower in delayed‐onset LN patients when compared to initial‐onset LN patients. Also, compared to initial‐onset LN patients, delayed‐onset LN patients had more relapses of LN, even with the addition of immunosuppressants. Patients included in this study were selected based on the guidelines for the treatment of LN;^4^, ^5^, ^6^ therefore, the available treatment options widely varied. In terms of induction therapies for LN, although more patients in the initial‐onset than those in the delayed‐onset LN groups were treated with mPSL pulse therapy, no significant difference was observed regarding the use of immunosuppressants between the 2 groups. Maintenance treatment for LN varied from case to case and included immunosuppressants, such as azathioprine, cyclosporine, TAC, mizoribine, and MMF. It was difficult to discuss the differences between different types of immunosuppressants because of the small number of cases that were administered each drug. In the present study, we were unable to describe the relationship between the use of immunosuppressants for maintenance treatment and the difference in relapse‐free rate between the initial‐ and delayed‐onset LN groups. Nakano et al reported a lower relapse‐free rate in the delayed‐onset LN group, but no difference was observed between the 2 groups in terms of the maintenance medications used;^18^ thus, this report may be informative. Ichinose et al previously reported that the early‐onset LN group was characterized by higher levels of anti‐dsDNA antibodies and hypocomplementemia with higher serological activity, and a lower index of chronicity compared to the late‐onset group.^22^ Park et al reported that glomerular sclerosis in the chronicity index was an independent predictor of complete remission after start of therapy in LN patients.^25^ In addition, it was difficult to calculate and compare the chronicity index due to the different historical backgrounds and organized evaluators. Comparing renal histological findings to the extent possible, in the initial‐onset LN group, 10 and 6 cases of global glomerulosclerosis and fibrous crescents, respectively, were observed among the 24 patients in the no‐recurrence group, whereas 3 and 1 cases of global glomerulosclerosis and fibrous crescents were observed among the 6 patients in the recurrence group. Conversely, in the delayed‐onset LN group, 2 and 1 cases of global glomerulosclerosis and fibrous crescents were observed among the 6 patients in the no‐recurrence group, whereas 3 cases of global glomerulosclerosis and fibrous crescents each were observed among the 7 patients in the recurrence group although the comparison was based only on the presence or absence of findings without considering differences in degree. In this study, we could not show the differences in chronicity findings between the initial‐ and delayed‐onset LN groups. Nakano et al stated that the relatively worse long‐term renal outcome in delayed‐onset LN was primarily because of failure to achieve sustained remission in these patients, and delayed‐onset LN might be a potential predictor of poorer treatment outcomes.^18^ Some reports state that a poor renal response to initial treatment and renal flares were strongly associated with future renal damages.^26^, ^27^, ^28^ It was thought that delayed‐onset LN patients were difficult to treat because they had already received immunosuppressive treatments for SLE before development of LN. As previously reported, one reason why delayed‐onset LN was hard to treat was that kidneys of delayed‐onset LN patients had more chronic damaged lesions compared to kidneys of initial‐onset LN patients. Therefore, delayed‐onset LN patients are considered to have a poor response to immunosuppressive therapies because immunosuppressive therapies are usually effective against active lesions of the kidneys, and not chronic damaged lesions. Medicines used before development of LN also might have influenced chronic damage of kidneys. It was also considered that some patients needed aggressive immunosuppressive treatments for extended periods because of active immune reaction since LN had newly developed despite prior treatment for SLE. The kidneys are among the main target organs of SLE. Jakes et al reported that renal involvement of SLE was observed in Asians, 21%–65% at diagnosis and 40%–82% over time.^29^ LN is an important factor that influences mortality in SLE.^3^ It was suggested that SLE patients without renal manifestation at disease onset should pay more attention to renal function and urinalysis in order to monitor development of LN. Since LN patients in this study were heterogeneous, this result was considered more useful in SLE patients.

On the other hand, we could not show any difference in relapse‐free rate between early and late remission patients as previously reported.^14^ One reason might be that renal histological background was varied in this study. Also, more LN patients of early remission had achieved remission by treatment with only PSL when compared to patients of late remission. It was suggested that LN patients of early remission might have included more patients with mild nephritis, and patients with early diagnosis and treatment with plasticity in renal lesions. However, patients of early remission who were treated with only PSL tended to experience recurrence of LN later. We were reminded of the importance of performing histological examination by renal biopsy as much as possible, and treating by adding immunosuppressants according to the algorithms of treatment of LN.

Despite the above, some study limitations were noted. First, renal biopsy was not performed in about 1/3 of the patients for reasons, such as high risk of bleeding complicated by APS, poor general condition, and refusal of examination. In clinical practice, because a number of LN patients had not undergone histological diagnosis of LN, it was relevant to include these patients. Second, due to the retrospective nature of the study, heterogeneity in LN patients could not be fully excluded, which has been reported as a limitation with retrospective observational studies on LN.^21^, ^22^, ^30^ Owing to the long study duration, the treatment regimen for LN varied based on time, and because many physicians treated patients in this study, treatment regimens were subtly different for each treating physician. Additionally, because we did not store serum samples from patients, we could not measure several biomarkers that are useful for determining the pathophysiology of LN. Third, the sample size was relatively small, especially the number of patients in late remission and delayed‐onset LN. Fourth, the histological diagnosis of the kidney did not follow the ISN/RPS classification for LN; therefore, some patients were diagnosed based on the World Health Organization's classification. Finally, it was difficult to define patients with delayed‐onset LN, and we defined such patients as those who were diagnosed with LN at ≥12 months after SLE diagnosis and who had already achieved remission of SLE before being diagnosed with LN. Although Jacobsen et al^16^ and Seligman et al^17^ reported that renal manifestation in most patients with SLE was observed within a year; several studies^15^, ^19^, ^22^ have defined delayed‐onset LN based on a time gap of 5 years between SLE diagnosis and LN development. Therefore, it could not be ruled out that the patients with delayed‐onset LN in this study might have included several patients with initial‐onset LN. Further, because the development of LN can sometimes be silent, that is, without an initial manifestation of proteinuria and hematuria, the inclusion of initial‐onset LN patients in the delayed‐onset LN group could not be ruled out. Therefore, we must focus on and interpret the results of similar studies reported from various countries, and it is necessary to conduct a prospective large‐scale, multicenter international collaborative study to verify the findings described here.

In conclusion, our study demonstrated that the relapse‐free rate was significantly higher in the initial‐onset LN group when compared to the delayed‐onset LN group of LN patients of various histopathological backgrounds. These data suggest that delayed‐onset LN is a risk factor for relapse of LN. Therefore, it is important that SLE patients who are not complicated by LN also be carefully monitored regarding renal function and urinalysis. When LN develops, a renal biopsy should be conducted and immunosuppressive therapies commenced as soon as possible.

## CONFLICT OF INTEREST

The authors of this work have nothing to disclose.

## AUTHOR CONTRIBUTIONS

Conceptualization, ES, MYF, JT, YF, NM, MH, TA, SS, TK, KM; methodology, ES, MYF, and KM; validation, ES, and KM; formal analysis, ES, MYF, and KM; investigation, ES, MYF, JT, YF, NM, MH, TA, SS, and KM; resources, ES, HK, HW, and KM; data curation, ES; writing—original draft preparation, ES, and KM; writing—review and editing, ES, TK, and KM; visualization, KM; supervision, HK, HW, KM; project administration, KM; funding acquisition, KM All authors have read and agreed to the published version of the manuscript.
